# Transcutaneous Auricular Vagus Nerve Stimulation-Paired Rehabilitation for Oromotor Feeding Problems in Newborns: An Open-Label Pilot Study

**DOI:** 10.3389/fnhum.2020.00077

**Published:** 2020-03-18

**Authors:** Bashar W. Badran, Dorothea D. Jenkins, Daniel Cook, Sean Thompson, Morgan Dancy, William H. DeVries, Georgia Mappin, Philipp Summers, Marom Bikson, Mark S. George

**Affiliations:** ^1^Department of Psychiatry, Medical University of South Carolina, Charleston, SC, United States; ^2^Department of Pediatrics, Medical University of South Carolina, Charleston, SC, United States; ^3^Department of Biomedical Engineering, City College of New York, New York, NY, United States; ^4^Ralph H. Johnson VA Medical Center, Charleston, SC, United States

**Keywords:** transcutaneous auricular vagus nerve stimulation, transcutaneous vagus nerve stimulation, vagus nerve stimulation, vagus nerve stimulation, feeding, pediatric rehabilitation, hypoxic–ischemic encephalopathy

## Abstract

Neonates born premature or who suffer brain injury at birth often have oral feeding dysfunction and do not meet oral intake requirements needed for discharge. Low oral intake volumes result in extended stays in the hospital (>2 months) and can lead to surgical implant and explant of a gastrostomy tube (G-tube). Prior work suggests pairing vagus nerve stimulation (VNS) with motor activity accelerates functional improvements after stroke, and transcutaneous auricular VNS (taVNS) has emerged as promising noninvasive form of VNS. Pairing taVNS with bottle-feeding rehabilitation may improve oromotor coordination and lead to improved oral intake volumes, ultimately avoiding the need for G-tube placement. We investigated whether taVNS paired with oromotor rehabilitation is tolerable and safe and facilitates motor learning in infants who have failed oral feeding. We enrolled 14 infants [11 premature and 3 hypoxic–ischemic encephalopathy (HIE)] who were slated for G-tube placement in a prospective, open-label study of taVNS-paired rehabilitation to increase feeding volumes. Once-daily taVNS was delivered to the left tragus during bottle feeding for 2 weeks, with optional extension. The primary outcome was attainment of oral feeding volumes and weight gain adequate for discharge without G-tube while also monitoring discomfort and heart rate (HR) as safety outcomes. We observed no adverse events related to stimulation, and stimulation-induced HR reductions were transient and safe and likely confirmed vagal engagement. Eight of 14 participants (57%) achieved adequate feeding volumes for discharge without G-tube (mean treatment length: 16 ± 6 days). We observed significant increases in feeding volume trajectories in responders compared with pre-stimulation (*p* < 0.05). taVNS-paired feeding rehabilitation appears safe and may improve oral feeding in infants with oromotor dyscoordination, increasing the rate of discharge without G-tube, warranting larger controlled trials.

## Introduction

In the motor task of feeding, neonates are required to coordinate a complex and rapid sequence of sucking, swallowing, and breathing, all integrated with a typical respiratory rate of 40 breaths per minute. This requires advanced sensorimotor integration of muscles of the face, head, and neck with the myelinated vagal regulation of breathing and heart rate (HR; Porges, 1992; Portales et al., 1997; Suess et al., 2000; Porges and Furman, 2011). Feeding difficulty is the primary reason for delayed hospital discharge in preterm infants with brain dysmaturation or near-term/term infants with hypoxic–ischemic encephalopathy (HIE) who are otherwise clinically stable and ready for discharge (Adamkin, 2006; Lau et al., 2015; Jackson et al., 2016). This increases hospital costs and is associated with a negative impact on long-term neurodevelopment, particularly with receptive and expressive language deficits (Adams-Chapman et al., 2013; Malas et al., 2015). The current standard of treatment for infant oromotor dysfunction consists of occupational or speech therapists feeding infants by mouth (PO) once a day to encourage safe feeding while learning this motor skill. However, many infants do not show improvement by term equivalent age, even after many weeks of rehabilitation with therapists, and have a gastrostomy tube (G-tube) placed for adequate nutrition.

Difficulty learning the motor sequence for oral feeding may be due to brain injury from infection, ischemia, and dysmaturity (Huang et al., 2015; Ismail et al., 2017). This diffuse injury results in less myelination and fewer brainstem–cortical connections (Duerden et al., 2015; Rocha-Ferreira and Hristova, 2016) and may lead to reduced corticobulbar regulation of both vagal activity and the striated muscles of the face, head, and neck (Suess et al., 2000). Atypical neural maturation with prematurity or brain injury also leads to overactive sympathetic inputs into the autonomic nervous system combined with lower parasympathetic vagal tone and persistent brainstem dysmaturity (Heilman et al., 2012; Rocha-Ferreira and Hristova, 2016). Such reactivity and neural dysmaturation make coordinating and learning a complex motor task even more difficult, explaining why the feeding mechanism must be taught through feeding rehabilitation, when it should be a normal reflex.

With improved survival rates of more critically ill neonates, the national rate of G-tube placement has doubled from 2000 to 2012 (Hatch et al., 2018). Complications of G-tube placement and removal often lead to subsequent hospitalizations or procedures after discharge from the nursery (McSweeney et al., 2015; Khalil et al., 2017; Hatch et al., 2018). At the Medical University of South Carolina (MUSC), preterm infants who have not reached full PO feeds by 40-week gestational age (GA) and/or after 40 days of attempting PO feeds have a >90% chance of eventually needing G-tube implantation to achieve full enteral feeds (Ryan and Gehle, 2019). Any therapy that facilitates motor learning and enhances feeding skills would have a significant impact for infants who fail feeding rehabilitation.

Vagus nerve stimulation (VNS) paired with motor activity enhances neuroplasticity, facilitates cortical reorganization and neurogenesis, and improves motor function post stroke (Porter et al., 2012; Engineer et al., 2015; Dawson et al., 2016). Recently, a noninvasive form of VNS known as transcutaneous auricular VNS (taVNS) targeting the auricular branch of the vagus nerve (ABVN) has demonstrated activation of the vagal afferent and efferent networks (Kraus et al., 2013; Garcia et al., 2017; Yakunina et al., 2017; Badran et al., 2018a,c). In patients with limb impairment post stroke or brain injury, pairing taVNS with motor activation can enhance plasticity and improve functional motor recovery (Dawson et al., 2016; Pruitt et al., 2016; Redgrave et al., 2018). This human work extends the large animal literature that demonstrates pairing VNS with a behavioral intervention restores brain function (Hays et al., 2014a,b; Khodaparast et al., 2014, 2016). Therefore, both animal and adult human data support the likely efficacy of VNS-paired with motor rehabilitation.

We applied this model of taVNS paired with a motor behavior to neonates who have failed to learn the oromotor skill of feeding. We conducted a prospective, open-label trial exploring the use of once-daily taVNS-paired rehabilitation training to enhance oral feeding behavior in neonates with oromotor dyscoordination. We hypothesized that taVNS paired with bottle feeding may function in a similar mechanism by enhancing cortical plasticity in neonates with oromotor deficits, resulting in improved acquisition of the sensorimotor skill of feeding. With a favorable safety profile in adults and the ability to treat noninvasively at the bedside, taVNS is an attractive therapeutic option for neuromodulation therapies in this vulnerable population.

## Materials and Methods

### Study Overview

**Figure 1:**
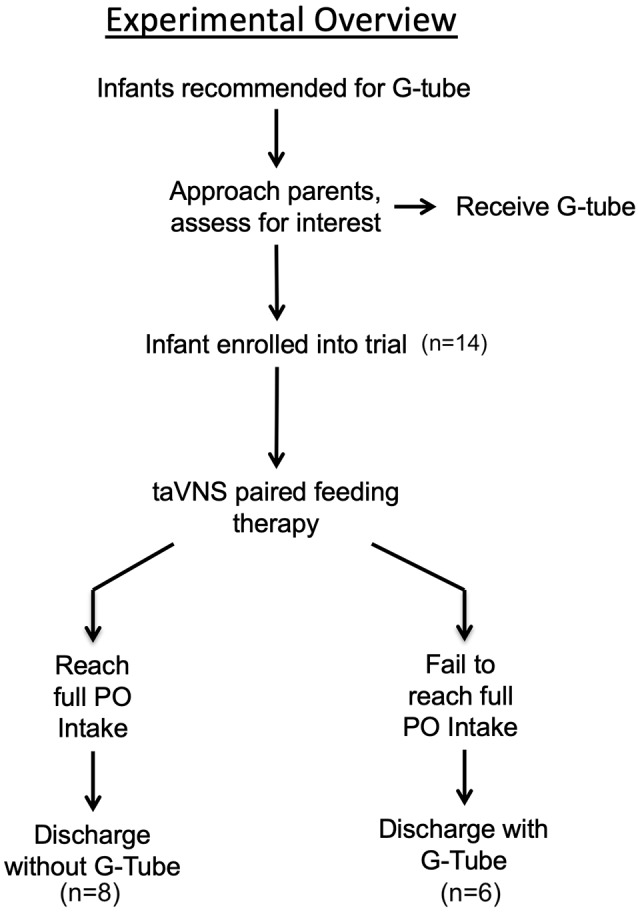
Experimental overview.

This study was conducted at the MUSC and was approved by the MUSC Institutional Review Board. After obtaining parental consent, we enrolled 14 participants who were consulted for G-tube placement in a prospective, open-label phase 0 trial to determine the feasibility, safety, and potential clinical benefit of a novel taVNS-paired oromotor rehabilitation paradigm in neonates with oromotor dyscoordination. We reported on five of the participants in this trial in an earlier brief communication (Badran et al., 2018b). Our primary clinical outcomes were improved PO feeding volumes and attaining full PO feeds adequate for discharge, thereby avoiding G-tube implantation (Figure 1).

### Participants

We included infants who were born premature at ≤33 weeks’ gestation at birth (*n* = 11) or suffered global HIE (*n* = 3) and who failed to make progress in PO volumes. Importantly, all enrolled participants were clinically determined to require a G-tube due to failure to achieve oral feeds sufficient for discharge from the hospital. Parents of all 14 infants had been approached about G-tube placement by the clinical teams prior to enrollment. Historically at MUSC, these infants would have <10% chance of avoiding a G-tube. We excluded infants who were clinically unstable, were unable to attempt every feed PO, were on significant respiratory support with frequent bradycardia or apnea events, or had cardiomyopathy.

### Transcutaneous Auricular Vagus Nerve Stimulation-Paired Feeding Protocol

We delivered taVNS once a day during a bottle feed, timed with observed sucking and swallowing for 30 min or the duration of the feed. Stimulation was paired with nutritive sucking and swallowing and was paused during rest or burping. The treatment period was 2 weeks, with the possibility to continue for an additional 2 weeks if substantial progress was made. If PO feeds had not progressed after 2 weeks of taVNS treatment, the parents and the clinical team made decisions about timing of G-tube placement.

#### Transcutaneous Auricular Vagus Nerve Stimulation Setup and Technique Refinement

**Figure 2:**
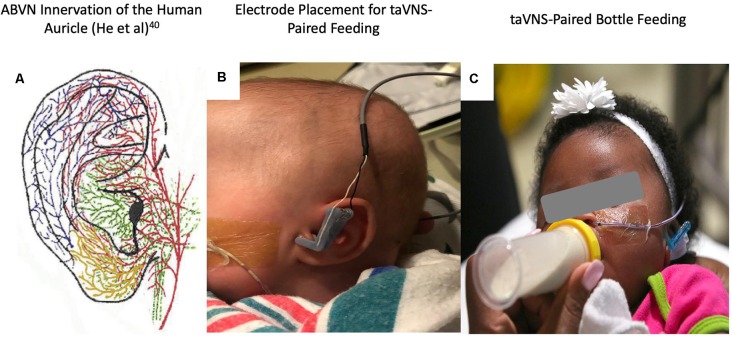
**(A)** Auricular vagus nerve fibers (He et al., 2012). **(B)** Close-up photo of the left ear with attached custom, 3D-printed transcutaneous auricular vagus nerve stimulation (taVNS) electrodes attached. **(C)** Photo of the taVNS-paired feeding session with stimulation delivered concurrently with bottle feeding (written informed consent was obtained from the legal guardians for the publication of this image).

We delivered taVNS using a constant current electrical nerve stimulator (Digitimer DS7AH, Digitimer LTD) connected to custom-designed neonatal ear electrodes (Figure 2). Electrodes targeted the anterior wall of the ear canal (anode) and the tragus (cathode). Stimulation was triggered manually for participants 1–7 or *via* a novel closed-loop electromyography (EMG) triggering system for participants 8–14 (Cook et al., 2020 under review, Brain Stimulation). The closed-loop trigger system was developed to more accurately pair stimulation trains with coordinated suck–swallow oromotor activation, to increase ease of use and to decrease operator tasks. Real-time EMG recordings were used to trigger taVNS stimulation based on masseter activation during suck–swallow. EMG leads were placed on the masseter muscle (recording), frontal eminence (reference), and center of the forehead (common).

We also refined the EMG-triggered pulse train for optimal pairing of stimulation with the sensorimotor sequence required for efficient feeding. This includes the pre-motor stage of sensing the nipple in the mouth, expressing and sensing milk on the tongue, and subsequent activation of multiple pharyngeal and hyoid muscles that effect swallowing. Many of these muscles are innervated by branches of the vagus nerve. With a 3-s pulse train following the EMG trigger, sucks that occurred at the end of the taVNS train did not receive stimulation (*n* = 4 participants). By lengthening the pulse train to 10 s, we achieved better pairing of stimulation with suck bursts (*n* = 3 participants).

#### Transcutaneous Auricular Vagus Nerve Stimulation Dosing

Stimulation parameters were as follows: frequency −25 Hz, pulse width −500 μs, and current intensity −0.1 mA below perceptual threshold (PT). We determined PT by increasing the stimulation current in 0.1-mA increments while monitoring for indication that the infant perceived the stimulation, indicated by shrugging, change in facial expression, or fidgety movements. A neonatologist and a technician performed the stimulation. During treatment, infants were fed by occupational or speech therapists, staff, or parents. A custom MATLAB program recorded pulses and current intensity delivered during each session. We recorded PO volume intake during taVNS feed, total daily PO volume, and any adverse events.

### Safety Monitoring and Target Engagement

The neonatal and infant pain scale (NIPS) scores (Lawrence et al., 1993; Witt et al., 2016) were recorded at initiation, midway, end, and 5 min after each treatment session. If NIPS scores increased greater than three points or the infant appeared to be sensing the stimulation, we decreased the current intensity by 0.1 mA. We monitored redness and skin irritation at electrode site and HR on bedside monitors for bradycardia, defined per nursery protocol as <80 bpm for 5 s. For target attainment, we recorded the lowest HR within the first 60 s of stimulation, the time to the lowest HR, and the rebound HR, to verify target engagement of vagus nerve using the parasympathetic response as an indicator (Badran et al., 2018c). We also recorded HR in 60-s epochs during taVNS-paired feeds and non-stimulation (control) feeds.

### Primary Outcome Measures

The primary safety outcomes were bradycardia events and NIPS score increase of greater than or equal to 3 points due to taVNS stimulation. The primary clinical outcome of this study was a binary endpoint of full oral feeds or G-tube implantation. Responders were participants who were able to increase and maintain full daily PO intake for 4 days (>120 ml/kg/day) and weight gain adequate for discharge (>20 g/day). Infants who received G-tubes for inadequate intake after taVNS treatment were classified as non-responders. Other outcomes were rate of increase of daily oral feeding volumes and length of time to achieve full oral feeds.

### Statistical Analyses

We analyzed group HR effects that compared HR measured before taVNS (or control) feed, the lowest HR at onset of taVNS (or control) feed, and during taVNS-paired feeding (or control) feeds using a one-way ANOVA. We then investigated the within-individual changes in HR using a paired *t*-test to compare each participant’s baseline and the lowest HR prior to feed to the lowest HR during PT within taVNS or control feeds, and unpaired *t*-tests for HR differences across taVNS or control feeds. Behavioral feeding data were analyzed by comparing the slopes of the linear regression generated from the rate of daily PO volume in two different time periods: (1) the 30 days before taVNS; and (2) taVNS-paired feeding period. We compared both between- and within-group subjects in a 2 × 2 design (pre/post taVNS and responder/non-responder). Videofluoroscopic swallow study (VFSS) scores prior to taVNS treatment initiation were compared with treatment period according to response group *via* unpaired *t*-test.

## Results

### Demographics

We enrolled 11 preterm and 3 near-term/term HIE infants. Clinical characteristics are noted in Table 1. Central nervous system (CNS) insults were prevalent (11/14) and consisted of intraventricular hemorrhage (IVH) or cerebellar hemorrhage, white matter infarction or periventricular leukomalacia (PVL), lenticulostriate vasculopathy (LSV), and acute moderate-to-severe HIE. A majority of infants (9/14) had sepsis complicating their neonatal course, which is associated with white matter neuroinflammation and infarction, and worse neurodevelopmental outcomes (Alshaikh et al., 2013; Bakhuizen et al., 2014; Bright et al., 2017; Dubner et al., 2019).

**Table 1:** Infant demographics.

taVNS-treated infants	Preterm (*n* = 11)	Term HIE (*n* = 3)
Sex M/F	5/6	0/3
Mean GA at birth (weeks)	28 ± 3	36 ± 0.5
Mean birth weight (g)	1,027 ± 453	2,600 ± 697
Mean GA at enrollment (weeks)	45 ± 5	40 ± 2
Mean days attempting PO before taVNS	57 ± 22	24 ± 10
Sepsis (including NEC, pneumonia, UTI, viral infections)	9	0
CNS abnormalities	8	3
IVH or other intracranial bleed (grade)	6 (grades 1 and 2)	1 (grade 3)
HIE (term HIE stage 2 *n* = 1), 3 (*n* = 1); preterm HIE stages not validated)	2	2
White matter infarction or PVL	2	1
Lenticulostriate vasculopathy	1	1
Infants of diabetic mothers	3	1
Hypoglycemia	4	1
Hyperglycemia	4	0
Gastroesophageal reflux requiring treatment	8	0
Aspiration on MBSS	5	1

*Note. GA, gestational age; NEC, necrotizing enterocolitis; UTI, urinary tract infection; PVL, periventricular leukomalacia; taVNS, transcutaneous auricular vagus nerve stimulation; HIE, hypoxic–ischemic encephalopathy; GA, gestational age; MBSS, modified barium swallow study*.

PO feeds were attempted for a mean (SD) of 49 ± 24.3 days before study enrollment in these 14 preterm and HIE infants. At study entry, most preterm infants were more than 44-week GA, well past term equivalent age, and had been trying to learn to feed for more than 40 days, at which point >90% of preterm infants at MUSC have attained full PO feeds (Ryan and Gehle, 2019). Prior to enrollment in this research trial, the clinical team had approached all parents about the need for a G-tube (Figure 1).

Thirteen out of 14 infants had clinical studies of videofluoroscopic barium swallow (VFSS, *n* = 11) or an impedance probe (*n* = 2) prior to enrollment. Six infants also had upper gastrointestinal (UGI) contrast studies. Eight infants had gastroesophageal reflux documented on one or more of these studies and were treated with histamine or proton pump antagonists. The VFSSs were performed and scored by three pediatric speech language pathologists using the Rosenbek scale (Rosenbek et al., 1996). Mean (SD) penetration and aspiration scores were 6 ± 3 with thin liquids (range 1–8). Six infants had maximum scores of 8, indicating aspiration below vocal folds with no attempt to eject liquid: three of these infants were trialed with thickened feeds prior to beginning the study; two infants continued to attempt with thin maternal breast milk, which could not be adequately thickened during the taVNS treatments; one infant showed dramatic improvement in oral feeding volumes to 100 ml/kg/day after 2 weeks of taVNS treatments but had persistent coughing during feeds and was transitioned to thickened feeds near the end of the treatment course.

### Safety

We monitored for bradycardia during both PT and during the stimulation-paired feed. There was only one bradycardia adverse event during a taVNS-paired feeding, likely unrelated to the stimulation as it was associated with choking and emesis, and readily rebounded with pausing the bottle feed.

There were no episodes of tragus irritation or redness at the electrode site. Discomfort with stimulation remained low, with the median NIPS scores [interquartile range (IQR)] of 0 (0, 1.0) before, during, and after the taVNS-paired feeding. Out of a total of 228 taVNS-paired feeding sessions, there were 10 sessions (4.3%) during which NIPS scores increased greater than or equal to 3 points from pre-stimulation to during taVNS-paired feeding. In seven instances, the fussiness resolved quickly, and in three instances (1.3%), stimulation current intensity was decreased for a persistent NIPS score increase. In 16 instances (7%), we decreased stimulation when we believed it was possible that the infant was feeling stimulation but did not demonstrate a change in NIPS score. Four feeds were stopped in one infant for excessive fussiness that did not resolve after stopping stimulation, related to reflux (pH probe was in place for one instance).

### Heart Rate as a Putative Biomarker of Vagus Target Engagement

**Figure 3:**
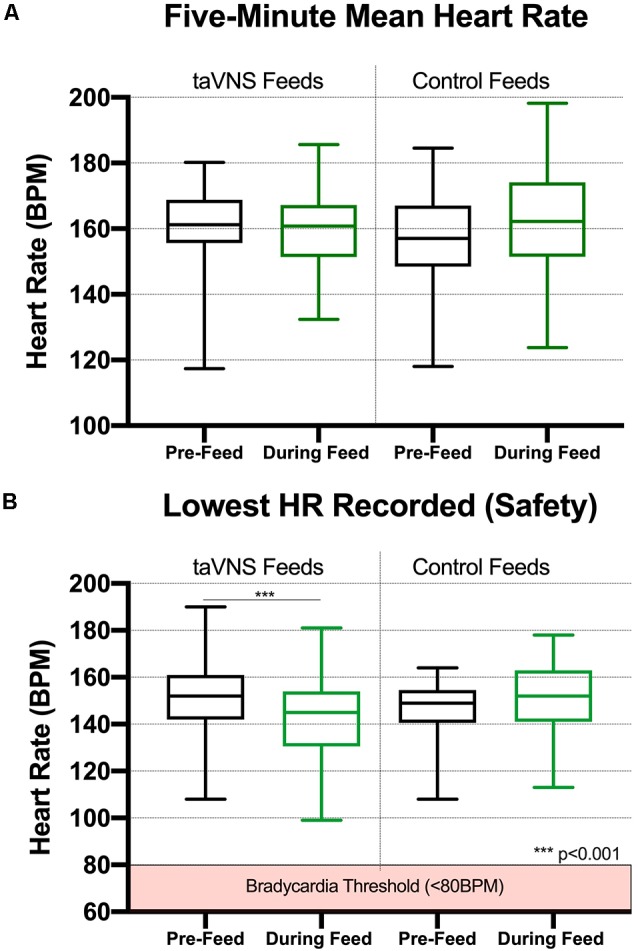
Box and whisker plots for heart rate (HR) data collected during taVNS and control feeds (min to max). **(A)** taVNS with these parameters induces immediate, safe reductions in HR that recover during feeding (*n* = 7, 39 total feedings in seven participants). For the 5-min epochs prior to perceptual threshold (PT) and during taVNS-paired feeding, HR was averaged in 60-s intervals for a total of 5 min. **(B)** The mean lowest HR during PT was calculated from real-time HR monitor recorded during onset of stimulation to determine the PT. taVNS induced significant reductions in HR compared with those in pre-stimulation baseline (*p* < 0.0001); however, these reductions recovered to baseline levels immediately during the taVNS-paired feeding. There was no significant reduction in HR during control feeds (feeding HR recorded without taVNS administered, *n* = 7, 19 feeds).

We performed a detailed analysis of HR changes in seven consecutive participants (#8–14), averaging 60-s HR data over the 5 min prior to PT and for the first 5 min of treatment. The mean HR before taVNS, compared with the onset of taVNS, revealed non-significant differences in physiology resulting from stimulation. The mean HR during the first 5 min before taVNS-paired feeding was 161 ± 11.7 bpm, compared with 159.6 ± 11.22 bpm during feeding (*n* = 39 feedings, seven participants, Figure 3A). For control feeds without stimulation, mean HR similarly does not change as a function of feeding. The mean HR was 156.7 ± 15.27 bpm for 5 min before the feed and 162.5 ± 15.98 bpm for the first 5 min during the feed, both non-significantly different from taVNS feeds.

The lowest HR was inspected as an indicator of safety profile. We compared the lowest HR before feed and the lowest HR during feeds. For the seven patients during taVNS feeds, the lowest HR before feed was 151.3 ± 15.1, and the lowest HR during onset of taVNS was 142.3 ± 16.9 (*p* = 0.0005). For control feeds, the lowest HR prior to feed was mean (SD) of 146.2 ± 14.6, and the lowest HR during feed was mean (SD) = 152.5 ± 15.7, and we found no significant difference between timepoints (Figure 3B).

When determining the PT, we consistently observed a decrease in HR with onset of stimulation. The transient HR drop was so common and predictable that we checked impedance and the earlobe contact, and electrode position if no HR decrease was observed. During determination of PT, HR decreased a mean of 20.5 ± 10.6 bpm or 12.6 ± 6.5% of the pre-taVNS HR (*n* = 105 taVNS sessions). In contrast, during control feeds, the HR decreased from before feed to the lowest HR during feed by a mean of 3.9 ± 6.4 bpm (*n* = 19 feeds, *n* = 7 subjects). The HR decrease with onset of taVNS stimulation was significantly greater than the HR change with control feeds (*p* < 0.00001). The comparison of the lowest HR before feed and with the lowest HR with taVNS onset vs. control feeds yielded similar results (Table 3).

**Table 2:** Clinical condition and treatment characteristics by responders and non-responders.

taVNS-treated infants	Responders *n* = 8	Non-responders *n* = 6	*p*
Preterm (mean GA at birth, birth weight)	6 (27 weeks, 877 g)	5 (29 weeks, 1,107 g)	
Term HIE	2	1	
Male sex	3	2
Mean days attempting PO pre-taVNS	48 ± 29	49 ± 16	ns
Mean PO volume over 5 days pre-taVNS	52 ± 22 ml/kg/day	45 ± 26 ml/kg/day	ns
Mean # taVNS treatments	16 ± 6	17 ± 3	ns
Average mA current intensity	0.82 ± 0.2	0.75 ± 0.2	ns
Total pulses all treatments (10^5^)	2.9 ± 1.7	2.2 ± 0.5	ns
IDM	1	3	
GERD requiring treatment	4	4
VFSS: mean (SD) PAS scores	6 ± 3	4 ± 3	ns
Aspiration on VFSS	5	1	
Esophagitis	1	2	
Periventricular leukomalacia	1	2	
Lenticulostriate vasculopathy	0	2	

*Note. taVNS, transcutaneous auricular vagus nerve stimulation; GA, gestational age; HIE, hypoxic–ischemic encephalopathy; GERD, gastroesophageal reflux disease; VFSS, videofluoroscopic swallow study; IDM, Infants of diabetic mothers; PAS, Penetration-Aspiration scale*.

**Table 3:** Lowest HR for 5 min prior to and during onset of taVNS vs. control feeds (*n* = 7 subjects).

	Lowest HR before	Lowest HR during	
taVNS-Paired feed	151.3 ± 15.1	142.3 ± 16.9	*p* = 0.0005
Control feed	146.2 ± 14.6	151.3 ± 15.1	*p* = 0.2

*Note. HR, heart rate; taVNS, transcutaneous auricular vagus nerve stimulation*.

**Figure 4:**
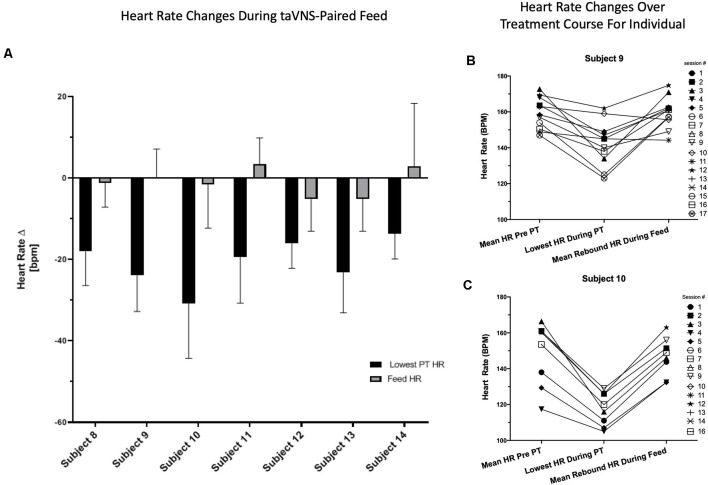
Reproducibility and reliability of individual HR change. **(A)** Individual HR change from baseline with onset of stimulation and during taVNS -paired feeding by individual subject. HR is averaged over all taVNS-paired feedings for each individual subject. **(B,C)** HR data from individual treatment sessions in two representative participants. HR changes are shown for each individual taVNS-paired feeding session over 5 min before and during taVNS-paired feedings and the lowest HR recorded during onset of stimulation with PT determination.

We observed this HR decrease as a rapid effect after taVNS onset. To determine the time frame of the HR changes, we recorded the time to the lowest HR and HR every 12 s during entire taVNS-paired feeds in three participants (*n* = 48 sessions). In these three participants, the mean HR decreased 16 ± 9 bpm or 10 ± 3% baseline HR within 26 ± 8 s of stimulation onset, followed by an HR rebound to or above baseline within 60 s from stimulation onset, which was maintained during the taVNS-paired feeding. These measurements were reproducible within and between individuals (Figures 4A–C), replicating our group’s prior HR findings in an adult human taVNS study (Badran et al., 2018c).

The HR decrease was likely due to vagus target engagement, as it was significantly different than the change in HR before and during control feedings without stimulation. During non-stimulation control feedings, the HR changed by a mean of −2.3 ± 14.0 bpm from HR before feed to the lowest HR during feed (*n* = 23, *ns*), compared with the rapid, transient mean HR decrease upon stimulation with taVNS (−20.5 ± 10.6 bpm, *n* = 104 feeds, *p* < 0.00001, *t*-test).

### Feeding Outcomes

Of the 14 participants enrolled, who had all failed to attain feeding after an average of 49 days trying (Table 1), eight infants attained full oral feeds with weight gain adequate for discharge from the hospital after a course of taVNS-feeding paired rehabilitation (responders), and six did not receive a G-tube (non-responders). This 57% response rate is higher than our institutional historical controls and published rates for preterm infants (Howe et al., 2007a,b; Jackson et al., 2016; Ryan and Gehle, 2019).

**Figure 5:**
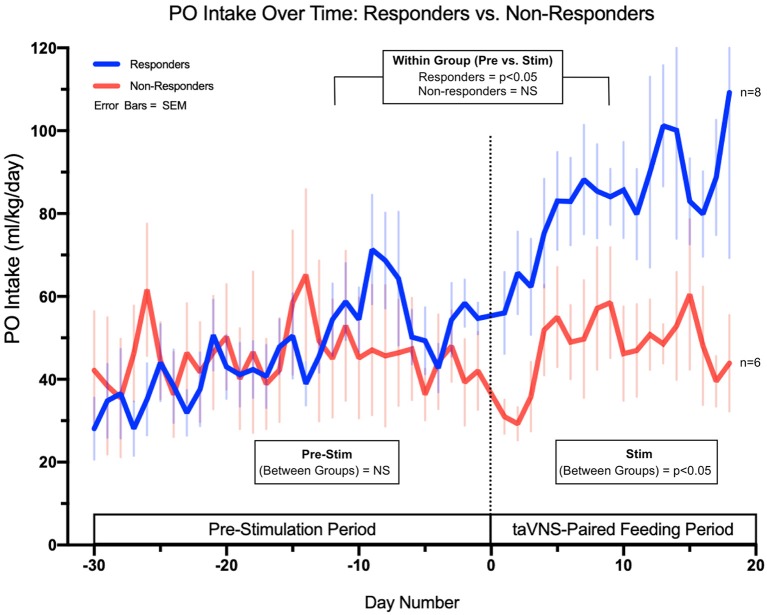
Daily PO intake feeding volumes in ml/kg/day for both responders (full PO feeds without G-tube) and non-responders (G-tube placement). The data demonstrate no significant difference in feeding trajectories between groups in the pre-stimulation phase but significant differences in trajectories between responders and non-responders upon initiation of taVNS-paired feeding.

We examined whether the responders were starting to improve oral feeds prior to enrolling in the trial. Although there is day-to-day variability in feeding volumes, the baseline rate of change of daily PO volume, averaged over 5 days immediately prior to taVNS treatment, was not significantly different between responders and non-responders (*p* = 0.15, Figure 5). With taVNS treatment, the rate of change of daily PO volume increased significantly in responders when compared with that in pre-treatment (*p* = 0.035). In non-responders, the mean rate of change of daily PO feeding volumes did not change from pre-treatment to during treatment (*p* = 0.29). Responders and non-responders did not differ in the number of taVNS treatments, average current intensity, or total pulses over all treatments (Table 2).

The VFSS scores prior to taVNS treatment were not different between response groups (*p* = 0.3). Among the six infants who demonstrated aspiration below the vocal cords without effort to eject the liquid, five were responders. Of the responders with aspiration, three were taking thickened feeds prior to starting the study, two continued on thin maternal breast milk feeds with pacing, and one infant on breast milk feeds was transitioned to thickened feeds during the taVNS treatment period, after making significant progress to 100 ml/kg/day but demonstrating persistent coughing.

## Discussion

In this phase 0 pilot trial, one taVNS-paired feeding per day was safe and well tolerated in infants who had failed to achieve full PO feeds and were referred for G-tube placement. Fifty-seven percent of treated infants were able to take all feeds by mouth within a mean of 16 days of treatment. taVNS-paired oral rehabilitation increased the likelihood of discharge without the need for G-tube implantation than did a historical retrospective comparison cohort who received standard feeding rehabilitation (Ryan and Gehle, 2019). A retrospective study of neonatal feeding outcomes at our institution built a predictive model that shows a minimal chance of reaching the required PO intake for discharge if the neonate has not reached 80 ml/kg/day by the 20th day attempting PO (Ryan and Gehle, 2019). Our cohort attempted PO for a mean of 49 ± 24.3 days prior to taVNS treatment, for which the in-house model predicts a spontaneous recovery rate of less than 10%.

Comparable published data indicate that preterm infants born at 25–32 weeks’ gestation, with respiratory complications of bronchopulmonary dysplasia, attained full oral feeds at 38.5 ± 2.8 weeks’ gestation, whereas those without BPD did so at 35.5 ± 1.7 weeks’ gestation (Howe et al., 2007b). In one large retrospective study of 560 preterm infants born at 32–36 week’s gestation, the median time to achieve full oral feeds was 12 days overall (Jackson et al., 2016). Although our taVNS-paired feeding study was non-randomized, our rate of successful attainment of full oral feeds is promising when compared with our historical control data as well as those from other observational studies.

Infants must develop oromotor skills of “suck–swallow” coordination in a particular sequence in order to feed effectively (Lau et al., 2003). Postmature infants (>41-week GA) who were born preterm and are not able to take full feeds by mouth may not be able to start oral feeds during a critical developmental window of oromotor neuroplasticity for learning feeding motor skills (Huang et al., 2015; Ismail et al., 2017). Sick newborns of term age (37- to 41-week GA) who have had critical illnesses frequently do not exhibit a suck–swallow reflex for feeding and may also have to learn this motor sequence, similar to preterm infants.

Both preterm and HIE infants suffer brain injury, triggering excessive stimulation of inflammatory pathways, impairing normal developmental functions of directing neuronal integration and foundational brain circuitry. After birth, the cortex and basal ganglia (BG) undergo significant integrative connectivity associated with shaping of central motor pathways. Disturbance of these processes leads to abnormal connections (Rocha-Ferreira and Hristova, 2016), and along with decreased populations of myelinating cells and inter-neurons, results in brain dysmaturity in preterm infants (Duerden et al., 2015) or overt brain injury in term HIE infants and subsequent motor, cognitive, and neurobehavioral impairments (Rocha-Ferreira and Hristova, 2016). Although postnatally the developing brain is more plastic than the adult brain and thus might be expected to have better recovery following injury, we engage the developing brain to a very limited extent with clinical rehabilitation therapy while infants are still in the nursery.

The data in both animals and adult humans are convincing in that VNS paired with a stimulus improves functioning (Porter et al., 2012; Khodaparast et al., 2013, 2014, 2016; Dawson et al., 2016; Capone et al., 2017; Meyers et al., 2018). In our neonates with brain dysmaturity or overt brain injury, neuromodulation of abnormal circuits may positively influence neuronal connectivity and neuroplasticity (Kilgard, 2012; Meyers et al., 2018). If we can influence the circuitry early, before motor patterns are fixed, we may improve the developmental deficits that these children experience, starting with feeding delays in the nursery. With approximately 380,000 preterm infants and 4,000 term infants with HIE born in the USA per year, this therapy may translate to a large number of infants and have major impact on their outcomes.

Decreasing G-tube placement and length of stay due to feeding delays is a significant, longer-term goal of these studies. The time to attain full feeds accounted for 90% of variance in length of stay in two reports (Adamkin, 2006; Jackson et al., 2016). Earlier discharge without a G-tube may reduce medical complications for the patients and decrease costs, while offering substantial benefit to families waiting to bring their children home. Beyond feeding and earlier discharge, reinforced plasticity of oromotor function may impact short- and long-term neurodevelopment, particularly language skills.

Even late preterm infants can have difficulty learning to feed. In one retrospective study, one third of 35–36 weeks’ gestation infants had feeding problems, and 76% of these had delayed discharge due to poor oromotor coordination (Wang et al., 2004). Also, males may have more difficulty as they have been shown to have later emergence of oral–lingual movements and pharyngeal activity than do females (Miller et al., 2006). In our study, we limited inclusion to preterm infants less than or equal to 33-week GA at birth or near-term to term infants with HIE. In the future, we need to enroll infants of wider GAs, conduct randomized controlled trials with sham stimulation, and enroll sufficient numbers for evaluation of sex differences.

Moreover, if taVNS is successful in the targeted motor behavior of feeding, we will extend investigation of early neuromodulatory therapy in high-risk infants to prevent or mitigate other life-long motor problems, such as cerebral palsy. This taVNS pilot trial, the first in human neonates, may provide a foundation for application of these therapies to infants at high risk for motor problems who have few alternative treatments.

Our HR data suggest that taVNS engages the vagal parasympathetic system. A rapid, transient HR decrease of 12% from baseline at the onset of taVNS stimulation was reproducible and reliable. The observed HR change suggests vagus nerve target engagement by taVNS-paired feeding in neonates and may be useful in multi-day trials.

Vagal efferent HR change is useful as a stimulation indicator if it is not harmful. Safety is the foremost consideration for treatments in vulnerable infants who are premature or have suffered HIE, who have often recovered from significant respiratory, cardiovascular, or CNS conditions. We did not observe adverse effects of bradycardia or rebound tachycardia that were solely related to stimulation, or any other adverse effects. Mean neonatal discomfort scores measured with the NIPS did not significantly increase during stimulation in over 200 treatment sessions, and we decreased current intensity in only three instances for possible stimulation-induced increase in NIPS scores. No parent withdrew their infant from the study, and parents and staff accepted the treatment as well as tolerated by the infants.

It is unclear why some participants did not respond. Systemic and neuro-inflammation or medications that inhibit synaptic plasticity and learning (e.g., mineralocorticoid receptor inhibitors) may impair learning this motor skill (Favrais et al., 2011; Kuban et al., 2014; Leviton et al., 2016; Kelley et al., 2017). Alternatively, other negative sensory inputs of reflux or esophagitis may counteract positive sensorimotor circuit stimulation (Wingenfeld and Otte, 2019). Half of the non-responders (3/6) and only one of eight responders were infants of diabetic mothers with poor glucose control during pregnancy, which induces a pro-oxidative state for the mother and fetus known to be associated with immune activation, endothelial cell injury, and worse fetal and neonatal outcomes (Teodoro et al., 2013; Durga et al., 2018). A larger sample size may identify specific predictors for responder status leading to a decrease in variance in treatment response.

Further investigation and refinement of treatment parameters will likely also improve treatment response. Although these stimulation parameters were partially optimized in adults and to some extent in this trial, the potential responsiveness of specific circuits is likely determined by the sum as well as timing of stimulatory and inhibitory impulses. For example, we initially tested a pulse-train length of 3 s with the EMG closed-loop system, which proved too short for rhythmic suck–swallow sequences in some infants. We then increased to a 10-s train with each EMG-driven stimulation.

## Limitations

The taVNS methodology was adjusted over the course of this study to more closely pair stimulation with feeding (Cook et al., 2020 in press *Brain Stimulation*). In participants 1–7, researchers activated taVNS manually when the infant was seen to be actively feeding; in participants 8–14, an EMG triggered taVNS-paired stimulation to sucking motor function. The latter would be expected to have better feeding outcomes, if learning depends on the precision of timing the stimulation with the motor activity. Over the study, we also developed several different ear electrodes to maximize contact and minimize the need for readjustment during feeds. All were confirmed by HR and resistance tests to deliver current to the tragus. We did not perform VFSS on every baby and did not explore the physiologic impairments in swallowing or causes of penetration/aspiration in this pilot trial. In future studies, we intend to document specific changes in swallowing function before and after taVNS-paired feeding treatment using a more precise scale adapted for infants (Martin-Harris et al., 2019). We also did not perform sham stimulation, to compare the lowest HR during sham and active taVNS stimulation. However, the HR changes with feeds in the control group indicate that HR usually increases during feeding, compared with the rapid decrease seen with PT in the taVNS feeds.

## Conclusion

This is the first study investigating taVNS paired with the motor sequence of suck, swallow, and breath to potentially enhance oromotor learning in neonates and infants. In infants who had failed oromotor rehabilitative feeding techniques by therapists, taVNS treatments resulted in 57% achieving full oral feeds adequate for discharge without needing a G-tube. Further, taVNS appeared safe in neonates and infants with no adverse effects. Target engagement may be determined at each session by a brief HR decrease. Further investigations and a randomized trial are needed to confirm our promising results of improved feeding outcomes in infants treated with taVNS.

## Data Availability Statement

The datasets generated for this study are available on request to the corresponding author.

## Ethics Statement

The studies involving human participants were reviewed and approved by MUSC IRB 1. Written informed consent to participate in this study was provided by the participants’ legal guardian/next of kin.

## Author Contributions

BB, DJ, DC, MD, WVD, GM, PS, ST, MB, and MG all made substantial contributions to the conception and design, acquisition of data, or analysis and interpretation of data and participated in drafting, editing, and final approval of this manuscript.

## Conflict of Interest

BB, DJ, DC and MG have pending patents on the methods described in this manuscript.

The remaining authors declare that the research was conducted in the absence of any commercial or financial relationships that could be construed as a potential conflict of interest.
